# Walking the walk: a case study of partnering with patients in designing and delivering a patient and public involvement implementation plan

**DOI:** 10.1186/s40900-026-00851-2

**Published:** 2026-02-12

**Authors:** Annabelle South, Kate Sturgeon, Asiyya Tahsin, Richard Stephens

**Affiliations:** 1 Medical Research Council Clinical Trials Unit at UCL, Institute of Clinical Trials and Methodology, UCL, London, UK; 2 Patient Advocate, Stevenage, UK

**Keywords:** Patient and public involvement, Clinical trials, Governance, Strategic PPI, Monitoring, Evaluation, Impact, Embedding, Culture

## Abstract

**Background:**

Clinical Trials Units (CTUs) in the UK are required to integrate patient and public involvement (PPI) into their research and operational strategies to meet national standards and registration criteria. While PPI in individual clinical trials is increasingly documented, strategic-level involvement within CTUs remains underreported. The Medical Research Council Clinical Trials Unit (MRC CTU) at University College London (UCL) has embedded PPI across its organisational strategy, partnering with PPI representatives to co-develop and deliver annual PPI implementation plans aligned with a five-year communications strategy.

**Description of process and outcomes:**

Since 2016 the MRC CTU at UCL has collaborated with its PPI Group, made up of staff and PPI representatives, to design and execute annual implementation plans that operationalise strategic PPI objectives. The 2024/25 plan included 18 activities, ranging from training and guidance development to evaluation and dissemination. All activities involved PPI representatives, with three led by them. Progress was monitored through predefined indicators and discussed in quarterly meetings. By year-end, 12 activities were fully achieved, five were underway, and one was postponed. Notable successes included high satisfaction with training sessions and the release of a podcast featuring PPI members. Challenges included delays in finalising a new participant information sheet template as a result of extensive internal review. The introduction of role descriptions for PPI representatives improved transparency and engagement for all parties.

**Conclusion:**

The MRC CTU at UCL’s strategic, co-designed approach to PPI fosters genuine partnership and integration of public involvement across the Unit. Annual implementation plans, collaboratively developed and evaluated, ensure alignment with long-term objectives and responsiveness to challenges. This model demonstrates how sustained, cross-cutting PPI can be effectively embedded in research infrastructure, contingent on core funding support. The approach offers a replicable framework for other CTUs aiming to enhance the impact of their strategic PPI.

**Clinical trial number:**

Not applicable.

**Supplementary Information:**

The online version contains supplementary material available at 10.1186/s40900-026-00851-2.

## Abstract (plain language summary)

This paper explores how patients are involved in decision-making at a strategic level in an academic clinical trials unit. It looks at how working together with patients helps the unit achieve its broader goals for patient and public involvement (PPI) beyond individual trials, and how working together supports the unit’s communications strategy. It also looks at how patients help track progress and judge results. The Medical Research Council Clinical Trials Unit at University College London is a large academic clinical trials unit delivering studies in cancer, infectious diseases and neurodegenerative diseases in and beyond the UK. For over twenty years it has involved patients in its research, starting with individual studies and gradually expanding to other areas like reviewing trial protocols. In 2016 the PPI group (made up of staff and PPI members) started to design and deliver annual plans to embed PPI in all the unit’s activities beyond the trials themselves. The 2024-25 unit PPI implementation plan had 18 activities, including training, writing guidance and sharing knowledge. PPI members were involved in all activities, and led three of them. The PPI group measured how well the plan was put into practice. This paper describes a model for long-lasting PPI that is built in effectively to the research organisation. It offers a framework for other CTUs aiming to enhance the value of their strategic PPI, and for PPI members seeking to widen their influence and involvement.

**Supplementary Information:**

The online version contains supplementary material available at 10.1186/s40900-026-00851-2.

## Background

Clinical Trials Units are specialist units that focus on designing, conducting, analysing and reporting clinical trials. To gain UK Clinical Research Collaboration registered clinical trials unit status, trials units must demonstrate that patient and public involvement and engagement is integrated into their research strategy, operational strategy, infrastructure, senior leadership and publication activities [1]. The UK Standards for Public Involvement include involving the public in research management, regulation, leadership and decision making [2]. There is a growing literature documenting approaches to and impact of patient and public involvement (PPI) in clinical trials [3–7], and many research funders and ethics committees require PPI as part of funding and ethics applications [8, 9]. Little has been published on the topic of PPI in governance and strategy of clinical trials units, as opposed to PPI in individual trials; a recent systematic review of reported PPI practices identify no papers relating to this [10].

The Medical Research Council Clinical Trials Unit at UCL is a large clinical trials unit running large, complex trials in infectious diseases, cancer and neurodegenerative diseases, both in the UK and around the world. We have a policy that all our clinical studies involve patients and the public, and some examples of our PPI approaches in individual studies have been published previously [3, 11–14]. Since 2016 we have worked in partnership with PPI representatives on our PPI group to develop our Unit’s 5-year Communication Strategies (which include PPI), and to develop, deliver and evaluate our annual PPI implementation plans of activities working towards the objectives set out in the strategy.

The MRC Clinical Trials Unit at UCL PPI Group is responsible for strategic oversight of the Unit’s PPI work, carries out cross cutting activities that are relevant across studies, and provides support and guidance to study teams. It does this through monitoring the compliance with the Unit PPI policy and identifying support needs; developing templates, tools and guidance documents; providing advice; training and teaching; and documenting, evaluating and sharing best practice. The group was originally established as the Cancer Consumer Group in 2006/7 and has since changed its name and scope to reflect the research portfolio of the Unit. The group includes both staff working in different roles and disease areas and PPI representatives with lived experience of the different disease areas as this is essential to ensure the work of the group is relevant and appropriate to the diverse trials portfolio of the Unit. This is set out in the terms of reference of the group. The PPI Group currently comprises 17 members, including 10 members of staff from across the functional groups and research themes of the Unit. Table 1 gives a breakdown of the current composition of the group. More information about the members of the group can be found on the MRC CTU at UCL website [15]. The 7 PPI representatives have lived experience of the diseases the MRC CTU at UCL works on, connections to patient advocacy and support groups, other skills that may be relevant to PPI activities (e.g. teaching and training experience), and some have experience of involvement in individual clinical trials. PPI representatives for the group are recruited from patient groups we work with and/or PPI contributors working on studies run by the Unit. It meets three times a year, with meetings attended by an average of 5 PPI representatives and 7 staff. People who are unable to attend a meeting can contribute via email, comments on draft documents and input to sub-group activities. All PPI representatives are paid expenses and an honoraria for their time, following the NIHR guidance on payments for public contributors [16].

**Table 1:** Composition of the MRCCTU at UCL PPI group

	PPI representatives	Staff
**Total**	7	10
**Sex**		
Female Male	4 3	9 1
**Age group**		
18–30	2	2
31–45		5
46–60	2	3
61+	3	
**Ethnicity**		
Asian British / Indian		1
Black African	2	
British Bangladeshi		1
Hispanic		1
Latin American	1	
White British	4	6
White Irish		1
Disease area*		
Cancer Infections Neurodegenerative diseases	3 4 1	7 6 3
**Role (staff only)**		
Administration Clinician Clinical Project Manager Communications Officer Epidemiologist Mixed methods researcher PPI coordinator Trial Manager		1 3 1 1 1 1 1 1

* Individuals may fit more than one category

The strategy objectives are at the Clinical Trial Unit level, rather than relating to individual studies. This case study describes the process we use for designing and delivering the patient and public involvement implementation plans. It uses the 2024-5 year as an example of this process, which has been in place since 2016, as this is the most recently completed year of the Implementation Plan at the time of writing. The GRIPP2 Short Form checklist can be found in Annex 1.

## Main text

### Developing the communications strategy and PPI implementation plans

The PPI Group inputted to the development of the 5-year Communications Strategy through discussion at meetings and comments on draft documents. Changes made as a result of this input included amending draft objectives; adding a section on values; and adding a section describing how PPI works across the CTU (including a diagram to visualise this). The Communications Strategy sets out aims and objectives for PPI, our PPI values, the audiences, how PPI is implemented at different levels within the Unit, and the resources available for PPI.

Our PPI Aims for 2021–2025 were:

- PPI is an integral part of the culture of the MRC CTU at UCL.
- The MRC CTU at UCL is regarded as a leader in the field of PPI nationally and internationally.

Our PPI objectives for this period were:

1. All our clinical studies involve patients and the public in ways that are appropriate for the study and add value to the research.
2. We explore the potential of PPI in methodology research around clinical trials and systematic reviews.
  - Can PPI add value to Methodology research, and, if so, in what circumstances?
  - How can we effectively involve patients and the public in these more abstract and technical research topics?
3. We learn from the PPI work we are doing through evaluation and sharing knowledge within the Unit.
4. We share our knowledge and experience beyond the Unit.

Each year the PPI Group draws up an implementation plan setting out how the Unit will work towards the objectives identified in this strategy. Annual implementation plans allow us regular opportunities to reflect on progress and be responsive to the changing needs of the CTU, while not being too onerous to develop. The Implementation Plan includes the activities we will undertake over the course of the year towards those objectives, who will lead on those activities and who will be part of their team (if applicable). It also includes monitoring and evaluation indicators. The group members discuss the plan, comment on drafts, and agree the final version. Once the Implementation Plan is agreed, the leader of each activity develops role descriptions setting out the desired experience and skills, and time commitment required for those who wish to be involved in that activity. These are shared with the group, and people can volunteer to be part of the activity sub-groups that interest them.

Ahead of each subsequent PPI group meeting a document with updates on progress is circulated, and activities with significant progress or challenges are discussed. Towards the end of the implementation plan year we produce a document setting out how we have performed against the monitoring and evaluation indicators. This is discussed in a PPI Group meeting to inform the development of the next year’s implementation plan, although as noted above, monitoring and evaluation are a continuous process.

### PPI implementation plan for 2024/5

Activities included in the PPI implementation plan for 2024/2025 included 18 discreet activities, all of which involved PPI representatives, and three of which were led by PPI representatives (activities 1.4, 4.2 and 4.3). The activities included a mix of continuing support (such as reviewing PPI plans, providing training and advice) and time-limited projects (such as evaluating the Methodology PPI panel, and releasing a podcast episode on the Unit’s PPI work). Alongside each activity were listed deliverables or indicators determined by the group to allow us to evaluate progress and measure success. Table 2 shows the activities planned against each objective, the deliverables/indicators, a summary on whether it was achieved within the year, and notes on the success (or otherwise) of the activity.

**Table 2:** Implementation plan activities and evaluation

Objective	Activity	Deliverables / M&E indicators	Achieved?	Notes
1. All our clinical studies involve patients and the public in ways that are appropriate for the study and add value to the research	1.1 Support the implementation of the new Standard Operating Procedure (SOP) through reviewing PPI plans, providing training & advice to staff	• Log of number of PPI plans reviewed • Number of people who attend SOP training • Evaluation forms from trainees	Yes	16 PPI Plans and 12 PPI annual review forms reviewed. 6 people attended SOP training, 90% found it useful.
	1.2 Provide and promote an advice service for study teams and patient representatives that includes consideration of the context of the study and the study population	• Log of contacts to the advice service	Yes	Regular emails from 7 patient reps on PPI Group. Support requested from methodologist to run focus group and help run stakeholder meeting. Supported requested from methodologist and clinician to help run focus groups for each of their projects.
	1.3 Deliver PPI Induction training for PPI representatives	• Number of attendees • Feedback collected via survey	Yes	8 registered participants. Average evaluation form ratings for sessions: • Introduction to clinical trials: 4.5/5 • Defining trial questions: 5/5 • Trial Designs: 5/5 • What happens during trials: 4.5/5 • Interpreting clinical trial results: 4.33/5 • Reporting clinical trial results: 4/5
	1.4 Launch the updated template Participant Information Sheet and consent form template and guidance.	• Final version of updated template and guidance document agreed by key stakeholders within the Unit • Template shared internally and externally • Share widely beyond the Unit	Ongoing	Delayed by internal processes. Clinical Project Manager is revising the draft.
	1.5 Develop youth/child-friendly participant information sheet guidance	• Final version of template and guidance document agreed by key stakeholders within the Unit • Template shared internally and externally • Share widely beyond the Unit	No	Postponed until the adult participant information sheet and consent form templates are launched
	1.6 To review the formal complaints process for PPI reps on studies (PPI representatives to be involved in the review process)	• Review of complaints • Review of log of actions taken/outcomes • Report on lessons learnt from complaints	Yes	No complaints had been received. Process was reviewed and is continuing unchanged for now.
	1.7 To collect data on PPI reps’ characteristics (gender, age, ethnicity etc.) to get a baseline on diversity amongst PPI reps	• Summary report on the diversity of our PPI representatives	Ongoing	We had an initial meeting with group, and requested question set from DISTINCT project to help develop survey.
	1.8 Continue to send PPI newsletter out quarterly	• Number of people who sign-up to newsletter • Engagement with information in newsletter, e.g. training, opportunities advertised • Number of people who open newsletter email and click on links • Number of PPI-authored pieces per newsletter issue	Yes	40 people signed-up Average open rate: 72% Average click rate: 11% 1 PPI-authored piece in the last 3 newsletters
2. We explore the potential of PPI in methodology research around clinical trials and systematic reviews	2.1 Evaluate the Methodology PPI Panel	• Report on lessons learnt from PPI in methodology research and next steps	Yes	Feedback report circulated and evaluation completed Evaluation is informing PPI work in the new Centre of Research Excellence.
3. We learn from the PPI work we are doing through evaluation and sharing knowledge within the Unit	3.1 Promote the library of Good PPI Practice within the Unit	• Number of examples of good practice in the library • Log of lessons learnt	Ongoing	In the library there are: • 4 PPI plans • Youth participation standards • E-book on PPI in Research: Practical experiences from different settings There is a link to the library on the new staff intranet
	3.2 To look for funding sources for project looking at the unique features around PPI in trials with complex designs	• Student working on project as their PhD	Ongoing	We are working with a potential PhD candidate who plans to submit this as a doctoral fellowship proposal.
	3.3 Evaluate how effective our PPI is in the Unit	• Feedback collected via survey for the Unit PPI • Feedback collected by trial teams via NIHR impact logs and online surveys for PPI in trials	Ongoing	The sub-group have met and are developing the evaluation plan
4. We share our knowledge and experience beyond the Unit	4.1 Teach about PPI on Masters, Bachelors and intercalated Bachelors courses	• Number of teaching sessions delivered • Number of students	Yes	1 session on Bachelors: 88 students 3 sessions on Masters course: ICTM0006: 19 students ICTM0006 (live session): 12 students ICTM0001: 19 students 3 sessions on intercalated Bachelors course: 4 students
	4.2 Lead training for external people on PPI in clinical trials	• Number of training sessions delivered • Number of trainees • Evaluation forms from trainees	Yes	1 online session in Feb 2025: 18 participants
	4.3 Present our work at International Clinical Trials Methodology Conference	• Abstract submitted for presentation on PPI implementation plan	Yes	Abstract presented at the conference by RS
	4.4 Use the MRC CTU at UCL social media accounts as a medium through which to share our learning on PPI, sharing a PPI resource on Twitter at least once a month.	• Number of posts about PPI • Engagement with posts • Social media training for PPI Group	Yes	20 tweets about PPI across all social media platforms (averaging 1.67 per month). Total 162 engagements across all PPI posts (averaging 8.1 engagements per post). Social media training carried out for PPI group.
	4.5 Release a podcast episode exploring the Unit’s history of PPI activity, featuring PPI group members as guests.	• Podcast listens • Engagement with social media posts sharing episodes	Yes	83 podcast listens. Total 39 engagements across 4 posts across all social media platforms (average 9.75 engagements per post). Total 2,874 impressions across 4 posts across all social media platforms (average 718.5 impressions per post).
	4.6 Keep PPI information up to date in CTU website.	• Number of views of these webpages	Yes	Number of views on PPI pages/section: · PPI 859 · PPI Resources 793 · About our PPI group 258 · Our PPI strategy 123 Total 2033 Updated new PPI resources including links to PPI complaints procedure and digital expenses system

By the end of the implementation plan period, 12 activities had been fully achieved, 5 were still underway and 1 had been postponed as a preceding activity it was dependent on was not yet completed. Highlights included positive feedback received from attendees at training delivered to staff (90% found it useful) and PPI representatives (with average ratings per session ranging from 4 out of 5 to 5 out of 5). The most challenging activity in the implementation plan was the development of a new participant information sheet template and guidance. While the PPI group had developed a draft, and updated guidance has been released, the template itself was not signed off during the implementation plan period, as a result of multiple, lengthy rounds of review and comments from CTU staff requesting additional text to be added or restructuring of the information. This delayed the start of another activity which will follow-on from the completed template.

Having updates on each activity in the implementation plan circulated prior to each PPI group meeting allowed the group to keep oversight of the progress with all 18 activities, and focus our discussions on activities with particular challenges.

## Discussion

The MRC CTU at UCL’s approach to achieving its aim of ensuring PPI is an integral part of the culture of the Unit, and playing a leadership role nationally and internationally, is to work with PPI representatives on our PPI group to co-design our PPI strategy and annual implementation plans. The annual implementation plans set out the activities we will work on each year to work towards the strategy objectives, and how we will monitor and evaluate them. The PPI group reviews progress on the implementation plan at each meeting. The activities in the implementation plan combine to embed PPI as a genuine and effective partnership within the Unit’s culture and working practices. While there are sometimes delays and set-backs in specific activities, overall this strategic and collaborative approach to planning and carrying out activities has allowed us to make progress towards our objectives. Together we are talking the talk and walking the walk. Box 1 summarises our tips for others looking to implement a similar strategic co-designed approach to PPI.

**Box 1:** Recommendations for others looking to implement a similar strategic co-designed approach

• Adopting this strategic co-designed approach is a long-term endeavour – this paper is looking at the implementation plan for a single year as an exemplar, but the process is the outcome of 9 years of developing and refining the approach
• This approach is dependent on support from CTU leaders to ensure the culture of the CTU recognises the importance of PPI at a governance level, as well as within individual studies • Our PPI Group is a strategic governance group that involves staff and PPI representatives working in partnership to oversee PPI at an organisational level, rather than providing PPI input to specific studies. As such, it is important to include: - staff working across different roles and the diversity of studies carried out by the CTU - a blend of PPI representatives with lived experience of the different health issues and the skills, experiences and understanding needed for a governance role
• Having a PPI coordinator whose role includes supporting the work of the PPI group, and the PPI representatives involved, has been important to the success of our approach

There is often an assumption that if a group has a majority of researchers and a minority of patients then this creates a power imbalance. However, this is not something we feel has been a problem for us. The researchers who are members of the PPI group are not all of one simple category (some work unit-wide and others are trial-specific, some are operational and some are clinical, some are senior and others are early-career) and the PPI representatives too come from a variety of backgrounds, diseases, nationalities, cultures and experiences. So there are many imbalances in the group, but importantly there is no imbalance of respect, and the differences between group members enhance rather than inhibit the way the group works. Ultimately the group is responsible to the unit’s senior management, and it is they who will have any final say on priorities, directions of travel, funding and other resources. There will always be that kind of power imbalance in any organisation. This far all decisions have been reached by consensus, and the unit’s senior management have been happy to support the implementation plans developed by the PPI group, especially as the group has been realistic about what is achievable and what is needed to resource it. That may be unusual and it may not be permanent, but for now, it is a genuine partnership of several interested parties sitting around a table rather than two opposing sides glaring at each other across it.

One improvement in the process we have introduced in the last two years is, once the implementation plan is agreed, developing role descriptions setting out the desired experience and skills, and time commitment involved for each activity. This allows PPI representatives to better understand what is required and decide whether that is appropriate for them, when deciding which activities to volunteer for. This enhancement was suggested by the PPI representatives on the PPI Group, and it has improved the transparency of the process and clarified expectations for all parties.

This paper adds to the existing literature on PPI by showing how a CTU can work in partnership with patients and the public to develop a strategy and implementation plan for PPI that cuts across multiple studies, engaging with the culture of the Unit rather than a specific trial. The role of the PPI representatives on the group corresponds with the ‘consumer advocate’ role described in the framework described by Gunatillake and colleagues [17]. The PPI group described here is similar to the Health Research Centre Patient-Partner Strategic Committee described by Boutin et al. [18], although that committee serves a Canadian health research centre, which has a different role to a CTU, including funding decisions for proof-of-concept studies. They describe how their committee engages patients in strategic decisions and governance activities, such as contributing to the development of a strategic plan, as well as input to specific research projects. The strategic approach taken to embedding PPI in the culture of the organisation, including the use of standard operating procedures, setting clear objectives and metrics and monitoring progress against them, and having a PPI group has some similarities to the approach used by the Wales Cancer Research Centre [19].

While individual studies conducted by the MRC CTU at UCL budget for study-specific PPI activities, the activities carried out by the group are not specific to any individual trial; they are cross-cutting. This means that they are dependent on core funding being available for PPI activities. We have had this funding available because the leadership of the MRC CTU at UCL have prioritised supporting PPI activities through the core funding the Unit receives from the Medical Research Council (until April 2026). This includes funding to reimburse PPI representatives expenses and pay honoraria in line with NIHR guidelines for their time in PPI group meetings and working on activities included in the annual implementation plan. It also includes salary costs for a part-time PPI Coordinator who supports the committee and leads on some of the activities in the plan. Without this core funding, and support from the leadership of the Unit, it would be very difficult to support this strategic activity that benefits the culture of the CTU and adds to the effectiveness of PPI across the whole Unit. The work of the MRC CTU at UCL PPI group also benefits the wider research and PPI community, firstly through the training, guidance and other resources we have developed and share, including the PPI in Clinical Trials course, PPI induction pack template [20], other templates, films and podcast episodes available via our website [21]. Secondly, the group has wider benefits through the upskilling of both patient advocates and CTU staff involved, which is then shared with the other organisations and groups they work with.

## Conclusions

We partnered with PPI representatives on our PPI group to develop and deliver both our PPI strategy and annual implementation plan. This annual cycle of co-development of the implementation plan, co-delivery and shared evaluation ensures we maintain our focus on achieving strategic objectives, and provides transparency around progress and challenges. This genuine and effective strategic, cross-cutting partnership approach is dependent on the availability of core funding. As we have shown, it adds value to the work we do, but it does require resourcing. The approach offers a replicable framework for other CTUs aiming to enhance the impact of their strategic PPI.

## Supplementary Information

Below is the link to the electronic supplementary material.

Supplementary Material 1: Annex 1: GRIPP2 Short Form Checklist

## Data Availability

No datasets were generated or analysed during the current study.

## Abbreviations

CTU: Clinical Trials Unit
MRC CTU at UCL: Medical Research Council Clinical Trials Unit at University College London
PhD: Philosophy doctorate
PPI: Patient and public involvement
SOP: Standard Operating Procedure
UK: United Kingdom of Great Britain and Northern Ireland
