# Supplementary material for: Walking the walk: a case study of partnering with patients in designing and delivering a patient and public involvement implementation plan
Source: Res Involv Engagem. 2026 Feb 12;12:21. doi: 10.1186/s40900-026-00851-2 (PMC12895777; doi:10.1186/s40900-026-00851-2)
Supplement: Supplementary file 1 — Supplementary Material 1: Annex 1: GRIPP2 Short Form Checklist [file 40900_2026_851_MOESM1_ESM.docx]

# GRIPP2 short form checklist

| **Section and topic** | **Item** | **Reported on page No** |
| --- | --- | --- |
| 1: Aim | Report the aim of PPI in the study | 7 |
| 2: Methods | Provide a clear description of the methods used for PPI in the study | 6-9 |
| 3: Study results | Outcomes—Report the results of PPI in the study, including both positive and negative outcomes | 9-10 |
| 4: Discussion and conclusions | Outcomes—Comment on the extent to which PPI influenced the study overall. Describe positive and negative effects | 10-14 |
| 5: Reflections/critical perspective | Comment critically on the study, reflecting on the things that went well and those that did not, so others can learn from this experience | 10-14, Box 1 |
